# RIC-3 expression and splicing regulate nAChR functional expression

**DOI:** 10.1186/s13041-016-0231-5

**Published:** 2016-04-29

**Authors:** Yael Ben-David, Tehila Mizrachi, Sarah Kagan, Tamar Krisher, Emiliano Cohen, Talma Brenner, Millet Treinin

**Affiliations:** Department of Medical Neurobiology, Faculty of Medicine, The Hebrew University, Ein Kerem, P.O. Box 12271, Jerusalem, 91120 Israel; Department of Neurology, Hadassah Medical Center, Jerusalem, Israel; Department of Biochemistry and Molecular Biology, Faculty of Medicine, The Hebrew University, Jerusalem, Israel

**Keywords:** Acetylcholine, RIC-3, Protein maturation, Alternative splicing, Disordered protein, Nicotinic acetylcholine receptors (nAChR), Inflammation

## Abstract

**Background:**

The nicotinic acetylcholine receptors form a large and diverse family of acetylcholine gated ion channels having diverse roles in the central nervous system. Maturation of nicotinic acetylcholine receptors is a complex and inefficient process requiring assistance from multiple cellular factors including RIC-3, a functionally conserved endoplasmic reticulum-resident protein and nicotinic acetylcholine receptor-specific chaperone. In mammals and in *Drosophila melanogaster* RIC-3 is alternatively spliced to produce multiple isoforms.

**Results:**

We used electrophysiological analysis in *Xenopus laevis* oocytes, *in situ* hybridization, and quantitative real-time polymerase chain reaction assays to investigate regulation of RIC-3’s expression and splicing and its effects on the expression of three major neuronal nicotinic acetylcholine receptors. We found that RIC-3 expression level and splicing affect nicotinic acetylcholine receptor functional expression and that two conserved RIC-3 isoforms express in the brain differentially. Moreover, in immune cells RIC-3 expression and splicing are regulated by inflammatory signals.

**Conclusions:**

Regulation of expression level and splicing of RIC-3 in brain and in immune cells following inflammation enables regulation of nicotinic acetylcholine receptor functional expression. Specifically, in immune cells such regulation via effects on α7 nicotinic acetylcholine receptor, known to function in the cholinergic anti-inflammatory pathway, may have a role in neuroinflammatory diseases.

**Electronic supplementary material:**

The online version of this article (doi:10.1186/s13041-016-0231-5) contains supplementary material, which is available to authorized users.

## Background

Nicotinic acetylcholine receptors (nAChRs) are a diverse family of pentameric neurotransmitter-gated ion channels. A total of 17 nAChR subunits (α1-α10 subunits which contain adjacent cysteines required for ligand binding, β1-β4, γ, δ, and ε) have been identified in vertebrates; nine subunits (α2-α7 and β2-β4) express in the mammalian nervous system. These subunits co-assemble to generate a wide variety of mostly heteromeric, cation-selective channels with diverse properties and expression patterns. Several, mostly non-synaptic—as opposed to direct, fast excitatory transmission—roles of nAChRs have been studied; among them, modulation of neurotransmitter release by presynaptic nAChRs is their most prevalent and well-studied role in the CNS [10, 11, 24] as well as roles in non-excitatory cells including an important role in controlling inflammation of the α7 nAChR [4, 21].

Maturation of nAChRs is a complex and time consuming process [29]. Several proteins were shown to affect functional expression and properties of nAChRs, including RIC-3, which significantly affects functional expression of multiple nAChRs and the recently identified NACHO protein which like RIC-3 is an ER-resident chaperone which enables functional expression of α7 nAChR in mammalian cell lines and is required for its expression in hippocampal neurons [17, 29, 30, 33].

RIC-3 is conserved for sequence and function [18, 19, 29]. In humans [19] multiple transcripts were found which show differential expression. These transcripts encode for multiple isoforms including a long full length (RIC-3 FL) transcript encoding for two membrane spanning domains followed by a coiled-coil domain and a shorter isoform lacking the coiled-coil domains (RIC-3 TM) [19, 29]. Alternative splicing of RIC-3 is seen across evolution and has been studied in detail in Drosophila [22]. Specifically the short TM isoform is conserved in mammals and its expression is likely to have functional implications [2, 3]. An additional mechanism likely to regulate RIC-3 function is its quantity, as high RIC-3 expression was shown to be detrimental, possibly due to formation of non-functional nAChR subunit-containing aggregates [1, 27, 32].

Effects of RIC-3 are receptor specific: While RIC-3 was shown to promote maturation of four different nAChRs in *C. elegans*, and is likely to be essential for functional expression of α7 nAChR, RIC-3 had either positive or negative effects on the two neuronal nAChRs α3β4 and α4β2 depending on the experimental system [18, 19, 22, 33]. Many neurons express several receptor subunits; enabling assembly of several different neuronal nAChRs [16]. Thus, identification of mechanisms preferentially promoting or inhibiting functional expression of one receptor relative to others is of interest.

Here we examine effects of expression level of two conserved alternatively spliced RIC-3 isoforms, FL and TM, on the three neuronal nAChRs: α3β4 – best characterized for its vital role in the autonomic nervous system [34], α4β3 – the most common nAChR in the CNS, having high affinity for ACh, and mediating the effects of smoking [14, 25], and α7 – which uniquely preferentially forms homomers, is characterized by low-affinity ACh binding, is expressed widely, is highly permeable to calcium, and strongly affects inflammation in immune cells [20, 26, 31]. We show that for both isoforms RIC-3-to-receptor ratio strongly affects receptor maturation in a receptor-specific manner. Furthermore, we show that expression and splicing of *ric-3* is regulated in mice, providing a new mechanism for regulating nAChR expression.

## Results

### RIC-3-to-subunit ratio affects nAChR functional expression

Previous studies have found conflicting effects of RIC-3, inhibited vs. enhanced functional expression, on α3β4 and α4β2 nAChR functional expression [19, 22]. In order to explore the hypothesis that the RIC-3-to-receptor subunits ratio may play a role in RIC-3’s effect on functional expression, we conducted the first systematic analysis of the effect of the full length (FL) RIC-3 isoform on these nAChRs, using the heterologous expression system *Xenopus laevis* oocytes (for a description of mouse RIC-3 transcripts see Additional file 1: Figure S1).

**Fig. 1:**
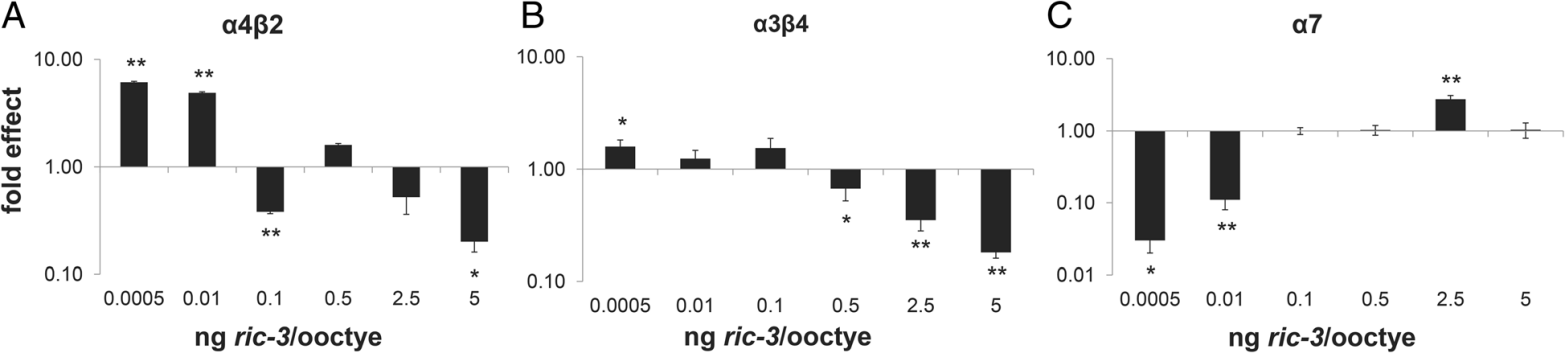
Effects of different amounts of FL on ACh-stimulated currents through each of three nAChRs: **a** α4β2; **b** α3β4; and **c** α7. Results were normalized to currents recorded in oocytes expressing the respective receptors in the absence of RIC-3 in the same experiment. Each bar represents 10–20 oocytes from 2 to 3 independent *X. laevis*. The y-axis ordinates are on a log scale. * indicates a p value of less than 0.05; ** indicates a p value of less than 0.01

We co-injected cRNA of FL *ric-3* with the cRNA of α4 and β2 subunits, or α3 and β4 subunits into *X. laevis* oocytes for electrophysiological analysis. Subunits were always injected at 5 ng/oocyte, while *ric-3* was injected in amounts ranging from 5 ng/oocyte to 0.0005 ng/oocyte. Our results (Fig. 1a and b) show current amplification at a relatively robust range of low to mid concentrations of *ric-3* for α4β2 and weaker less robust amplification for α3β4 at similar concentrations (0.0005–0.01 ng/oocyte and 0.0005–0.25 ng/oocyte, respectively). Inhibition was seen at only two concentrations checked for α4β2 (0.1 ng/oocyte and 5 ng/oocyte), while inhibition was more robust, spanning an entire order of magnitude, at the highest concentrations checked (0.5–5 ng/oocyte) for α3β4. For all receptors, not all concentrations tested are shown; a number of the lower concentrations produced very similar results and so only representative low and mid concentrations are shown.

In similar analysis of effects of RIC-3-to-α7 subunit ratio (Fig. 1c), co-expression of α7 with FL showed amplified currents at a narrow, high concentration of *ric-3* (2.5 ng/oocyte) and, for the first time, inhibition of currents was shown at low concentrations (0.0005–0.05 ng/oocyte). This novel finding of inhibition at low concentrations was robust, spanning two orders of magnitude. Interestingly, analysis of the effects of the FL human RIC-3 isoform showed no significant inhibition at any of the concentrations examined (Additional file 2: Figure S2). Results in Additional file 2: Figure S2 show inhibition by mouse FL RIC-3 at the highest concentration used, a result similar to previously described effects of high RIC-3-to-α7 ratio [1], but different from what is seen in Fig. 1c. Differences in the results shown in Fig. 1c compared to Additional file 2: Figure S2 are likely due to different cRNA quality or variability in protein expression between experiments.

### RIC-3 alternative splicing’s effects on nAChR functional expression

Alternative splicing of RIC-3 generates multiple isoforms [19, 22, 29]. Two conserved isoforms of RIC-3 are the FL isoform (isoform a) and the transmembranal (TM) isoform which includes the two transmembrane domains of the FL isoform but does not include the C-terminal coiled-coil domain [29]. This TM isoform (isoform d), unlike other alternatively spliced human isoforms [29], is also found in mice (for example, clone BU705427) [2] and in swine (FS667185). For a description of mouse *ric-3* transcripts found in databases of full length transcripts see Additional file 1: Figure S1. In humans this isoform is represented by several human EST clones from multiple tissues and its expression in mice is described below. To explore whether mammalian alternative splicing of RIC-3 – specifically, the presence or absence of the coiled-coil domain – affects RIC-3’s effects on nAChRs, we repeated the systemic analysis described above, this time, co-expressing the TM isoform together with nAChR subunits.

**Fig. 2:**
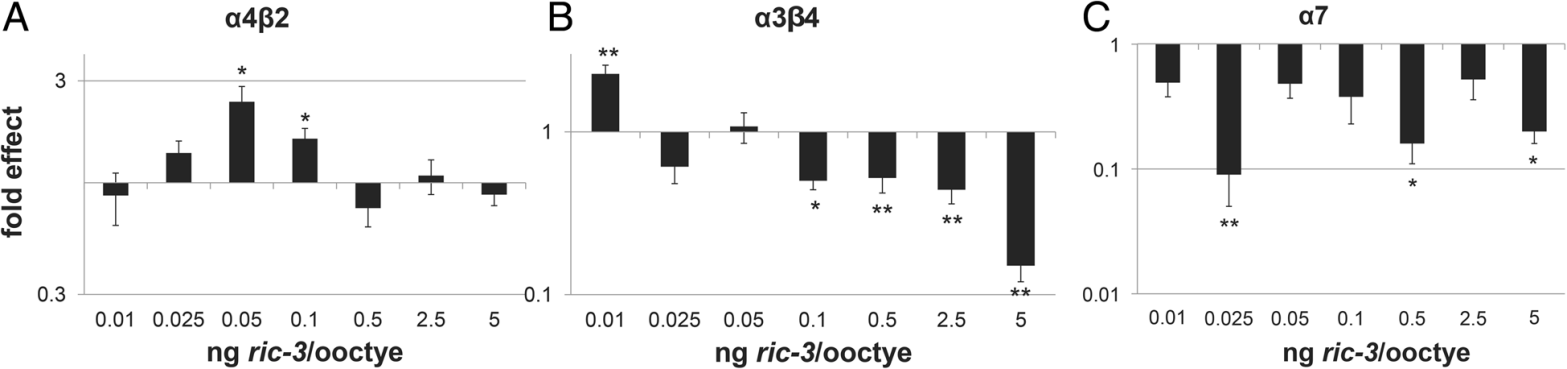
Effects of different amounts of TM on ACh-stimulated currents through each of three nAChRs: **a** α4β2; **b** α3β4; and **c** α7. Results were normalized to currents recorded in oocytes expressing the respective receptors in the absence of RIC-3 in the same experiment. Each bar represents 10–20 oocytes from 2 to 3 independent *X. laevis.* The y-axis ordinates are on a log scale. * indicates a p value of less than 0.05; ** indicates a p value of less than 0.01

While co-expression of α7 with FL showed amplified currents at a high concentration of FL RNA (Fig. 1c), co-expression of α7 with TM showed inhibition of currents at all concentrations (Fig. 2c). This is in contrast to α4β2 where currents were amplified or inhibited depending on the amount of FL expressed, while no significant inhibition was seen at any concentration of TM expression (Fig. 2a), or to α3β4 where currents were amplified at low concentrations of RIC-3 and inhibited at high concentrations, regardless of RIC-3 isoform (Fig. 2b).

**Fig. 3:**
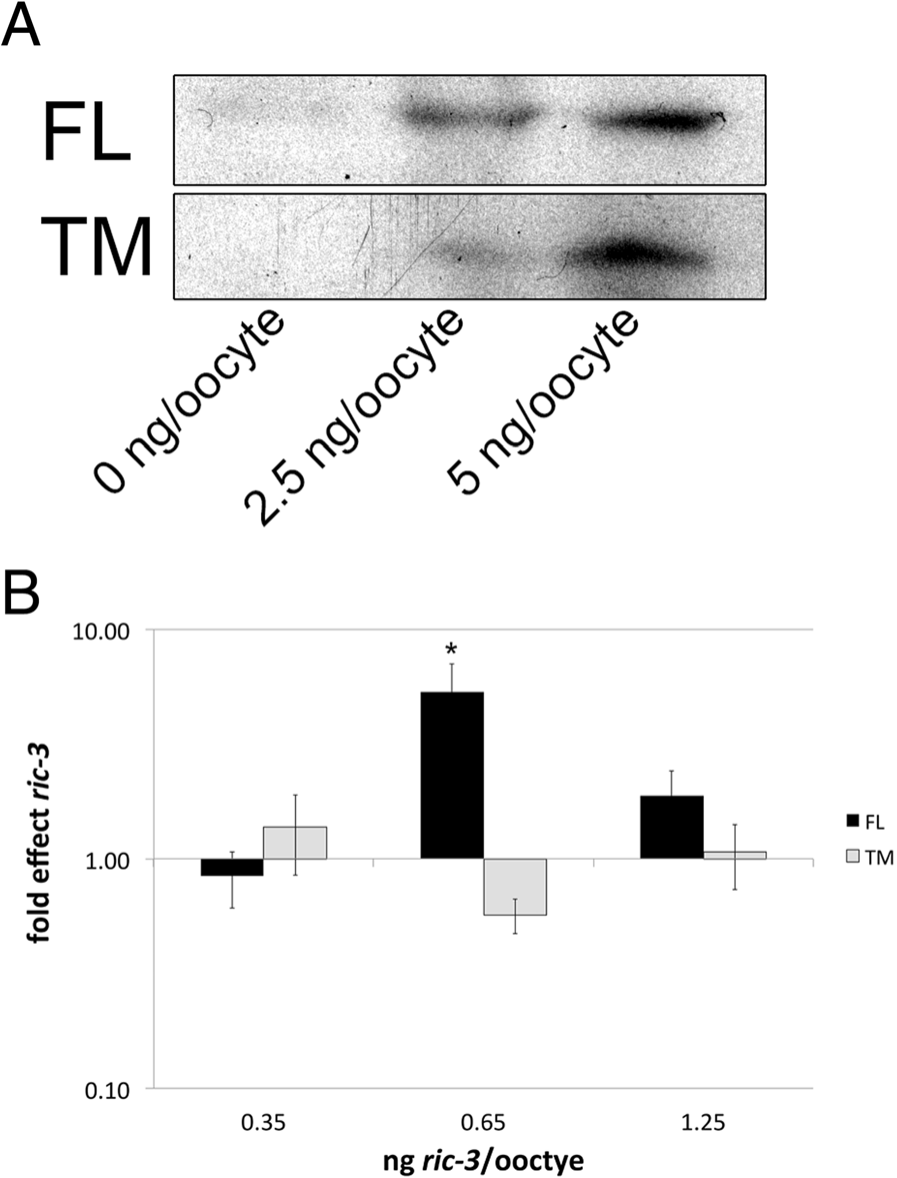
Effects of TM and FL cannot be attributed to expression level. **a** Representative Western Blot of the *myc-*tagged transcript-expressing oocytes showing similar expression of FL and TM. A single oocyte’s worth of homogenate prepared from an average of about 5 oocytes, was run in each lane; the Western was repeated twice with nearly identical results; **b** Electrophysiological recordings of ACh-stimulated currents through α7, co-expressed with different amounts of *myc*-tagged RIC-3 transcripts. Each bar represents 7–11 oocytes from 2 independent *X. laevis.* The y-axis ordinates are on a log scale. * indicates a p value of less than 0.05

As amplifying effects on α7 nAChR are seen at a single concentration of FL and no concentration of TM (Figs. 1c and 2c) we checked whether differences in expression of the two isoforms explain their different effects. To compare expression levels of these isoforms we tagged both isoforms with two copies of *myc* at the C-terminus. Using this tag we found that the two isoforms express similarly (Fig. 3a). Moreover, electrophysiology experiments show different effects of RIC-3 FL vs. TM in spite of having similar expression levels in the same experiment (Fig. 3). Therefore we can rule out protein stability or efficacy of translation as the mechanism of RIC-3 isoforms’ differential effects on nAChRs’ functional expression. We note that in these experiments, the concentration where amplification is seen is shifted relative to experiments with untagged FL (Fig. 1c). This is likely to be a result of differences in cRNA quality or of effects of the tag on protein stability (Fig. 3b relative to Figs. 1c and 2c).

### FL and TM isoforms of RIC-3 express in the brain

The different effects of the two RIC-3 isoforms examined and the effect of RIC-3-to-receptor ratio on functional expression of co-expressed nAChRs (Figs. 1 and 2) indicate that there is likely to be a physiological significance to mechanisms regulating alternative splicing and quantity of RIC-3. To explore the functional implications of alternative splicing in the mammalian brain we examined the expression patterns of the two isoforms in the brains of mice.

**Fig. 4:**
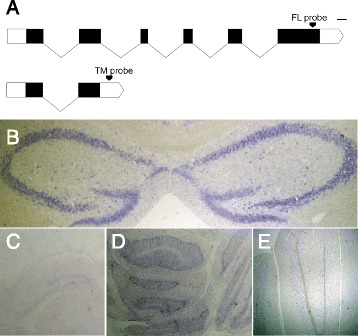
RIC-3 expression in mouse brain by *in situ* hybridization. **a** Schematic representation of the transcripts encoding the FL and TM isoforms and loci of the isoform-specific probes. Lines, introns, black filled bars coding sequences, white filled bars non-coding (UTRs). Scale bar, 100 bp, size of non-coding sequences is not to scale. **b** representative image of FL expression in hippocampus. **c** Representative image of TM expression in hippocampus. **d** Representative image of FL expression in cerebellum. **e** Representative image of TM expression in cerebellum

For this we used *in situ* hybridization using oligodeoxynucleotide probes consisting of approximately 250 nucleotides; these probes correspond to the antisense strand of unique 3′ untranslated regions of the FL and TM isoforms, and to an antisense strand region common of all known isoforms (positive control, overlapping the first two exons which are found in all known mouse transcripts, same as in [19]) and its sense strand (negative control) (Fig. 4a and Additional file 1: Figure S1). We found that both isoforms indeed express in the naïve mouse brain and that expression may be regulated. Specifically, while the positive control probe was clearly visible throughout the hippocampus and cerebellum (similarly to what was shown by [19]) and the negative control probe was not visible anywhere in the brain, the FL-specific probe showed a strong signal throughout the hippocampus, CA1, CA2 and CA3 and dentate gyrus (Fig. 4b) in all of the brains checked, and a somewhat weaker signal in the cerebellum (Fig. 4d) of two of the three brains. The TM-specific probe was visible in the cerebellum (Fig. 4e) of all brains but only very weakly in the hippocampus (Fig. 4c).

### FL and TM isoforms of RIC-3 express differentially

Results above suggested that RIC-3 alternative splicing is regulated. However, as *in situ* hybridization is a qualitative rather than quantitative assay, we further evaluated the extent of differential expression using qRT-PCR. For this we assessed expression of FL and TM in the dissected CNS regions of naïve young adult female mice.

**Fig. 5:**
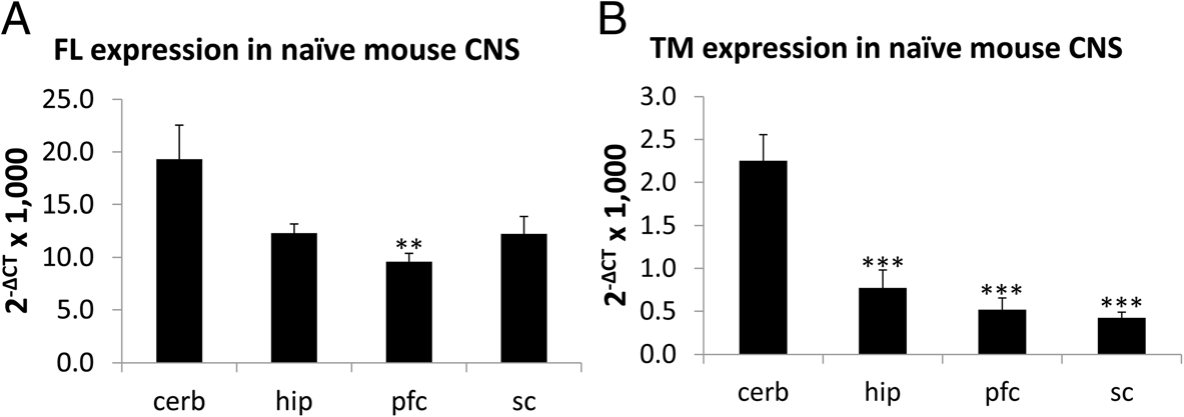
RIC-3 expression in mouse brain by qRT-PCR. **a** FL and **b** TM expression in CNS regions of naïve mice. ** indicates a p value of less than 0.01; *** indicates a p value of less than 0.001 according to Students t-test comparing expression in each tissue to expression in the cerebellum. Expression was tested using qRT-PCR for each isoform in each tissue of 10–12 mice. Expression values are normalized to GAPDH expression and multiplied by a factor of 1,000 for convenience. Cerb = cerebellum; hip = hippocampus; pfc = prefrontal cortex; sc = spinal cord

We found that FL expression is greater than TM expression in all regions investigated by an order of magnitude (compare Fig. 5a to b), and that the amount of each isoform expressed is significantly different in different tissues. Specifically, FL expression was highest in the cerebellum, followed by the spinal cord, hippocampus, and finally prefrontal cortex where the difference was statistically significant (*p* < 0.001) relative to cerebellum according to ANOVA (Fig. 5a). TM expression was also highest in the cerebellum, followed by the hippocampus, prefrontal cortex and finally, spinal cord, all three of which were lower significantly relative to cerebellum (*p* < 0.001) according to ANOVA (Fig. 5b). Interestingly, while FL expression was about 8.6 times greater than TM in the cerebellum and 8.3 times greater in the hippocampus, the difference was significantly greater in the prefrontal cortex (18.5 times greater) and in the spinal cord (29.0 times greater). Thus, both expression and splicing of RIC-3 are likely to be regulated in a brain region-specific manner.

### Expression level and splicing of RIC-3 in immune cells and following inflammation

This study found that effects of RIC-3 on α7 nAChR are strongly dependent on RIC-3-to-receptor ratio and on *ric-*3 splicing (Figs. 1c and 2c). Previous studies found that α7 plays an important role in immune cells [31]. Therefore, it was interesting to investigate regulation of *ric-3* expression and splicing in immune cells: mouse spleen cells and RAW264.7 (a mouse macrophage cell line), where α7 activation was previously shown to inhibit lipopolysaccharide (LPS)-dependent activation [31].

**Fig. 6:**
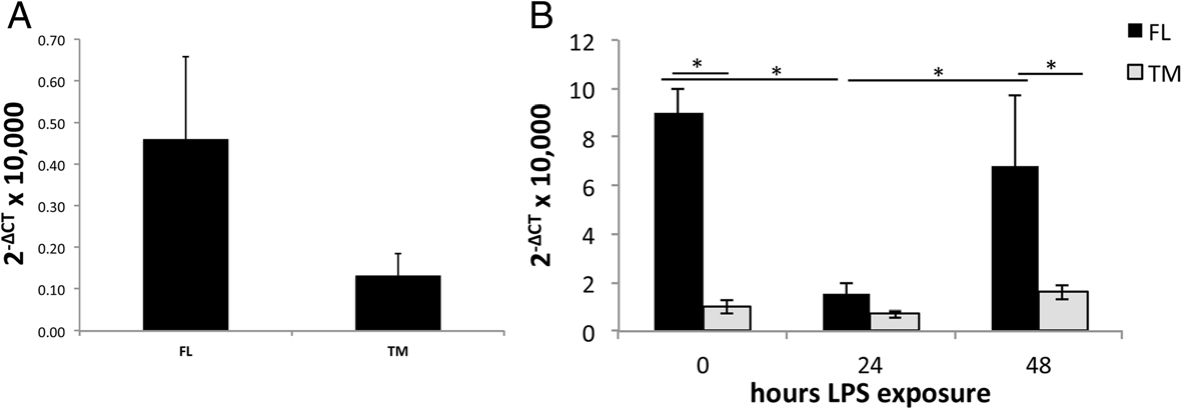
RIC-3 expression in **a** mouse spleen cells, and **b** RAW264.7 cells at 0, 24 and 48 h following exposure to LPS. * indicates a p value of less than 0.05; ** indicates a p value of less than 0.01 according to two-way ANOVA with Bonferroni correction. Expression was tested using qRT-PCR for each isoform in each of 6 spleens, or from each of three independent RAW264.7 experiments. Expression values are normalized to GAPDH expression and multiplied by a factor of 10,000 for convenience

Similar to in the CNS, FL expression was higher than TM in the spleens of naïve young adult female mice (Fig. 6a). Overall, *ric-*3 expression was about two orders of magnitude lower in the spleen relative to the CNS. In RAW264.7 cells, overall expression of *ric-3* was higher than in spleen cells but the FL to TM ratio was similar to spleen cells and brain regions (Fig. 6b, time zero).

To examine the effects of pro-inflammatory stimuli on *ric-3* expression, RAW264.7 cells were stimulated with 25 ng/ml LPS and collected after 0, 24 and 48 h exposure for RNA isolation, reverse transcription, and analysis by qRT-PCR. At 0 h, expression of FL was about 10 times great than TM expression (Fig. 6b). However, after 24 h exposure to LPS, FL expression was significantly decreased while TM expression was only mildly decreased, and after 48 h exposure FL expression almost returned to unexposed levels and TM expression also mildly increased (Fig. 6b). In other words, in RAW264.7 cells acute pro-inflammatory stimuli inhibited *ric-3* expression – far more significantly FL expression than TM expression – which recovered with prolonged exposure. Previous studies have shown pro-inflammatory effects of LPS after 48 h, and even 72 h; thus our results showing recovery of *ric-3* expression after 48 h are not likely to be a result of time limited effects of LPS or to degradation of LPS [9, 13, 35].

## Discussion

Taken together our results show that the effects of RIC-3 on nAChRs appear to depend on the RIC-3-to-receptor ratio and on the particular receptor subtype. These results reconcile previously conflicting reports of the effects of human RIC-3 on α4β2 and α3β4 nAChRs, as previous studies used a narrow range of RIC-3-to-receptor ratio and thus missed inhibitory or amplifying effects of RIC-3 [7, 19, 24]. The sensitivity to RIC-3 quantity is likely to be not only receptor-specific, but also species specific as no inhibition was seen of α7 nAChR when co-expressed with human RIC-3, in contrast to mouse RIC-3. In addition, by using a wide range of mouse RIC-3 concentrations we also saw inhibition of α7 nAChR at low concentrations for the first time; this inhibition may be due to a minimum ratio of RIC-3-to-α7 subunit being required for the amplifying mechanism of RIC-3 on α7 nAChR, while below a certain threshold the presence of RIC-3 is inhibitory; such inhibition may be explained by RIC-3’s binding to individual subunits preventing them from assembling on their own while not facilitating receptor assembly via coiled-coil domain dimerization, as suggested by Wang et al. [32]; dimerization which may require high RIC-3 concentration.

Structure function and bioinformatic analysis of RIC-3 suggested that it is a disordered protein [8]. Tompa et al. [28] described that some intrinsically disordered proteins elicit opposing (inhibiting and activating) action on different partners or even the same partner molecule even via the same site by adopting different conformations after binding, and that the amount of disordered protein expressed may be part of the mechanism for these opposing effects. Our results are, therefore, consistent with the suggestion that RIC-3’s functions depend on its disordered structure [8].

The current results also show that RIC-3 alternative splicing affects nAChR functional expression. Specifically, the coiled-coil domain may be uniquely necessary for RIC-3’s amplifying effect on α7; a result consistent with previously published results [32] showing that the coiled-coil domain is essential for RIC-3’s positive effects on α7 in cells that require RIC-3 for functional expression of α7. Moreover, our results suggest that the coiled-coil domain is necessary for the inhibitory effects of RIC-3 on α4β2 nAChR.

Together, our results suggest the regulation of RIC-3 expression and/or splicing is likely to have functional implication. Thus we explored the expression of the two conserved *ric-3* transcripts, FL and TM which have been analyzed here for their effects on neuronal nAChRs, in mice. *In situ* hybridization experiments with DIG-labeled probes allowed us to identify specific brain structures where each isoform expressed. However, while this method carries the advantage of high spatial resolution within brain structures, and general differences in signal strength were noted, it is not a reliably quantitative method and is biased to highlight cell-dense structures such as the hippocampus. In addition, variability of *ric-3* detection via *in situ* is evident by variability between mice in this study and when comparing the current *in situ* results to previously published results by our lab [19] and to those in the Allen Brain Atlas. Specifically, our previous analysis, unlike the current analysis, did not detect *ric-3* expression in the dentate gyrus [19]. In addition, the Allen Brain Atlas studies showed different partially overlapping expression patterns in two different mice (http://www.brain-map.org/search/index.html?query=%40entrez_id%20320360); these two brains were hybridized with different probes: one of which is to the 3′ untranslated region found only in the longer FL transcript (Additional file 1: Figure S1), and the other of which identifies all known mouse isoforms other than TM including the longer FL transcript.

To better analyze *ric-3*’s expression levels we used qRT-PCR which while having lower spatial resolution, as brain tissue must be dissected manually for experimentation, provides more accurate quantitative data which has been averaged over many animals. Taken together these findings support that regulation of RIC-3 expression level and alternative splicing in a region specific manner – co-localizing with different nAChRs – may enable region-specific regulation of nAChR functional expression. Particularly in neurons where expression of receptor subunits overlaps, mechanisms regulating RIC-3 expression level and/or splicing may preferentially promote or inhibit functional expression of one receptor relative to another.

Similarly, in immune cells, where the nAChR α7 plays a well-established role in the cholinergic anti-inflammatory pathway [4], we found that RIC-3 expression and splicing are regulated by a stimulus (LPS) inducing inflammation and that FL is the more strongly regulated isoform. These results together with the known dependence of α7 nAChR expression on RIC-3 and the known importance of α7 nAChR in regulating inflammation suggest that regulation of *ric-3* expression and splicing may have a role in regulating inflammation. Moreover, the very narrow range of concentrations at which mouse *ric-*3 enhances *α7* nAChRs functional expression enables strong effects of this regulatory mechanism either disinhibition if at resting levels of *ric-*3 its high expression inhibits α7 functional expression [1] or inhibition if resting levels enable its expression. As previous work showed upregulation of α7 nAChR expression in the same RAW264.7, immune cells, following the same pro-inflammatory stimuli the first possibility is more likely [23].

## Conclusions

We found that the effects of RIC-3 on nAChRs depend on the RIC-3 alternatively spliced isoform and on the RIC-3-to-receptor ratio; sensitivity to the latter in particular is both receptor-specific and RIC-3 isoform-specific. We also found that alternatively spliced isoforms of RIC-3 express in the brain differentially. Taken together we can propose a possible mechanism of RIC-3’s effect on nAChRs: That the splicing and amount of RIC-3 expressed create a complex mechanism for regulating the amplification or inhibition of nAChR functional expression. Furthermore, expression level and splicing of RIC-3 in immune cells and following pro-inflammatory stimulation suggests that this regulatory mechanism, via regulation of α7 nAChR function plays a role in regulating inflammation in a physiological setting and may have a role in neuroinflammatory diseases involving α7 nAChR.

## Methods

### Two-electrode voltage clamp

The mouse FL transcript (RIKEN clone A330090D22; NCBI locus BB195671) was acquired from imaGenes in the pFLCI vector, linearized with KpnI and transcribed in vitro using T7 RNA polymerase. Mouse TM transcript (RIKEN clone A230057E24; NCBI locus AK038724) was acquired from imaGenes in the pFLCI vector, linearized with KpnI and transcribed in vitro using T7 RNA polymerase. Rat α7 was inserted into pGEMH19, an oocyte expression vector having *X. laevis* globin untranslated regions, linearized with NheI and transcribed in vitro using T7 RNA polymerase. Rat α4 [15] in pSGEM was linearized with SfiI and transcribed in vitro using T7 RNA polymerase. Rat α3 [5] in pSP64 was linearized with EcoRI and transcribed in vitro using SP6 RNA polymerase. Rat β2 [12] in pSGEM was linearized with SfiI and transcribed in vitro using T7 RNA polymerase. Rat β4 [6] in pBS(SK-) was linearized with XhoI and transcribed in vitro using T3 RNA polymerase.

In vitro synthesis and injection of cRNAs into oocytes was previously described [2]. Briefly, in vitro transcribed and capped cRNAs were injected at final concentrations of 0.1 μg/μl for nAChR subunits and 0.1–0.00001 μg/μl for *ric-3* isoforms.

Macroscopic currents were recorded one to five days after injection, depending on the cRNA (1–2 days for α3β4 and α4β2 and up to 5 days for α7); comparisons for effects of *ric-3* FL or TM quantity on a specific receptor were all done on the same day. Cells were placed in a 2 ml bath that was perfused with medium and penetrated with two 0.5–1.5 MΩ 3 M KCl-filled glass microelectrodes attached to a GeneClamp 500B amplifier (Axon Instruments, Foster City, CA), using a two-electrode voltage clamp with active ground configuration and an HS-2A headstage (Axon Instruments). A Pentium 4 PC system employing the pCLAMP9 (AxoScope) software (Axon Instruments) was used for maintaining voltage clamp. Cells were clamped at −70 mV. Oocytes with leak currents <150 nA were used.

Electrodes were filled with 3 M KCl. The extracellular recording solution contained ND96 (96 mM NaCl, 2 mM KCl, 5 mM Hepes, pH 7.5) with 5 mM MgCl. The current and the voltage in the voltage-clamp circuit were recorded simultaneously and were saved directly onto the computer. 1 mM ACh agonist (Sigma-Aldrich) was prepared and used to stimulate oocytes. Results are presented as the mean ± SEM with n equal to the number of oocytes tested and N equal to number of different frogs in each experiment.

### Statistical analysis

Data analysis was conducted using Microsoft Excel after removing outliers per Grubb’s Outlier Test. To reduce noise due to variability in expression levels between different experiments, the current amplitudes were normalized to the average response for oocytes expressing only the receptor without *ric-3* in the same experiment. Student t-test and ANOVA were used to determine significance, as appropriate.

### Western blot

RIC-3 (FL and TM) constructs for protein quantification were prepared by tagging each transcript with two copies of the myc gene at the C-terminus using Q5® Site-Directed Mutagenesis Kit (New England Biolabs).

Oocytes were injected with 5 ng/oocyte α7 RNA and 0.35 ng, 0.65 ng, or 1.25 ng myc-tagged *ric-3* RNA. On the fifth day of expression, electrophysiology recordings were made and then oocytes were homogenized by repetitive pipetting in 25 μl/oocytes buffer (20 mM Tris-HCl (pH 8.0), 100 mM NaCl, 1 % Triton X-100) with Complete Mini, EDTA free, protease inhibitor mixture tablets (Roche Applied Science) (1 tablet/7 ml of buffer). After 30 min on ice, the homogenate was centrifuged at 4 °C. The supernatant was aliquoted, and sample buffer (125 mM Tris, 4 % SDS, 20 % glycerol, 0.2 % bromphenol blue, and 2 % β-mercaptoethanol) was added at 1:3 buffer-to-sample ratio.

Proteins were separated on 10 % SDS-PAGE, transferred to nitrocellulose membrane, and immunoblotted with polyclonal rabbit anti-myc 1:2000 (Abcam) followed by goat anti-rabbit HRP 1:20000 (Jackson Immunoresearch).

### *In situ* hybridization

The sequence of the oligodeoxynucleotide probes consisted of approximately 250 nucleotides. The probes correspond to anti-sense strands of unique 3′ untranslated regions of the FL or TM isoforms (Fig. 4a), the positive and negative controls corresponding to a region, encompassing the first two exons, common to all isoforms were previously described [19]. The probes were synthesized, briefly, by linearizing the vector-insert construct and using the resulting template for RNA synthesis using DIG-labeled ribonucleotides. Probes were purified using RNeasy Mini Kit for RNA cleanup (Qiagen) and quality assessment performed by agarose gel electrophoresis.

The protocol was repeated on the brains of three female ICR mice purchased from Harlan Laboratories, ranging from 10 to 21 weeks of age at the time of sacrifice. Brain tissue was fixed in 4 % paraformaldehyde, paraffin sliced, rehydrated, treated with proteinase K and refixed with 4 % formaldehyde.

Hybridization with DIG-labeled RNA probes was performed by dripping 100 μl prewarmed hybridization mix + 1 μl probe per slide and placed upright in a humid chamber containing Watman paper drenched with 50 % formamide solution and incubated overnight at 70 °C. Washed slices were then incubated overnight with anti DIG-AP antibody (90 μl/slide). On the third day slices were washed and developed, moved to PBS in the dark at RT for 3 h, mounted and imaged.

For the last hour of exposure, the slices were observed every 10–20 min, and exposure was stopped when background started to show uniform, ubiquitous, non-specific DIG signal. Brain structures with a clear DIG signal were considered positively expressing the probe target, and those with no significant signal-to-noise ratio were considered negative for probe target expression.

### Quantitative real-time PCR

RNA was extracted using Bio Tri Reagent (Bio-Lab, Ltd.), and single-stranded cDNA was synthesized from 2 μg total RNA using High Capacity Reverse Transcriptase Kit (Applied Biosystems, Foster City, CA, USA).

The expression of FL and TM was quantified using the StepOne Plus Real Time PCR System (Applied Biosystems). For each 20 μl qPCR reaction volume, 10 μl Fast SYBR green master mix (Applied Biosystems), primers at concentrations chosen in calibration experiments (all from IDT – Integrated DNA Technologies), 1 μl cDNA (50 ng) and DDW were mixed together. qPCR was performed at 95° for 20 s followed by 40 cycles of 95° for 3 s and then 60° for 30 s, followed by the melt curve stage. All reactions were run in triplicate. Data were normalized to the reference gene GAPDH. All raw data cycle thresholds were well within the range of reliable detection.

The primers used were as listed below, at final concentrations of 500 nM GAPDH and 100 nM RIC-3 isoforms; signal threshold was set at 0.8. For statistical analysis, raw C_T_ at threshold was recorded, normalized to GAPDH, and final expression was calculated as 2^-ΔCT^.

1. GAPDH forward: CTC TGC TCC TCC CTG TTC CA
2. GAPDH reverse: CTG GCA CTG CAC AAG AAG ATG
3. FL forward: AAG CCA CCA AGG AAA ACT TGC C
4. FL reverse: GCT ATG GTA TTG AAC ACA GAG GAT GCA GAG G
5. TM forward: GCG ATG GAT TTG AAA GGG CTA CAT GA
6. TM reverse: CCA GAT CAT TCC AAT CTA TGG CTT TGG

### Ethics approval and consent to participate

All animal tissue was acquired and handled in accordance with the guidelines for the ethical use of animal models of the Authority for Biological and Biomedical Models, The Hebrew University of Jerusalem.

### Consent for publication

Not applicable.

### Availability of data and material

In the absence of a suitable public repository for the datasets supporting the conclusions of this article, raw data for Figs. 1, 2, 3, 5 and 6, in Microsoft Excel format, can be supplied upon request.

## Additional files

Additional file 1: Figure S1. Mouse ric-3 isoforms according to sequences of full length transcripts. Shaded - ORF, black & white lines – hydrophobic regions (the first hydrophobic region is likely to function as a signal peptide), black coil-coiled domain. Empty rectangle - 3’ UTR. Intron size is not to scale. Asterisk marks site of an extra amino acid (serine) in the edited transcript. 1) AK138461 encoding for the FL isoform. 2) AK134663 similar to the FL isoform but edited and with a longer UTR; it is unknown if all edited transcripts have longer 3′ UTRs. 3) AK082275 and AK053760. 4) AK038724 encoding for the TM isoform. (PDF 165 kb) Additional file 2: Figure S2. Effects of FL human RIC-3 vs. mouse FL on α7 nAChR functional expression in *X. leavis* oocytes. Results were normalized to currents recorded in oocytes expressing the respective receptors in the absence of RIC-3 in the same experiment. Each bar represents 10–20 oocytes from 2 to 3 independent *X. laevis*. The y-axis ordinates are on a log scale. * indicates a p value of less than 0.05; ** indicates a p value of less than 0.01. (PDF 714 kb)

## Abbreviations

ACh: acetylcholine
cerb: cerebellum
CNS: central nervous system
FL: full length RIC-3 isoform
hip: hippocampus
KO: knock out
LPS: lipopolysaccharide
nAChR: nicotinic acetylcholine receptors
pfc: prefrontal cortex
sc: spinal cord
TM: transmembrane domains only RIC-3 isoform
