# Supplementary figures and images for: RIC-3 expression and splicing regulate nAChR functional expression
Source: Mol Brain. 2016 Apr 29;9:47. doi: 10.1186/s13041-016-0231-5 (PMC4850696; doi:10.1186/s13041-016-0231-5)

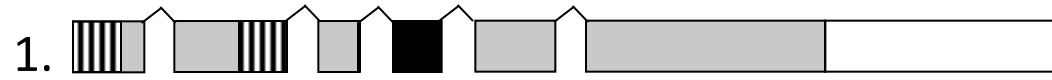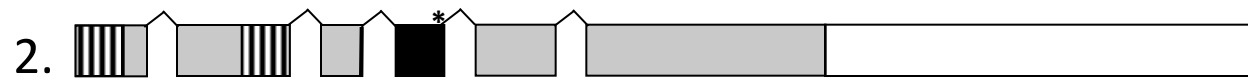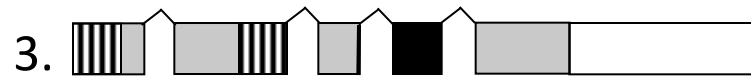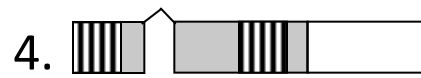

Supplement: Additional file 1: Figure S1. — Mouse ric-3 isoforms according to sequences of full length transcripts. Shaded - ORF, black & white lines – hydrophobic regions (the first hydrophobic region is likely to function as a signal peptide), black coil-coiled domain. Empty rectangle - 3’ UTR. Intron size is not to scale. Asterisk marks site of an extra amino acid (serine) in the edited transcript. 1) AK138461 encoding for the FL isoform. 2) AK134663 similar to the FL isoform but edited and with a longer UTR; it is unknown if all edited transcripts have longer 3′ UTRs. 3) AK082275 and AK053760. 4) AK038724 encoding for the TM isoform. (PDF 165 kb) [file 13041_2016_231_MOESM1_ESM.pdf]

**Fold effect**

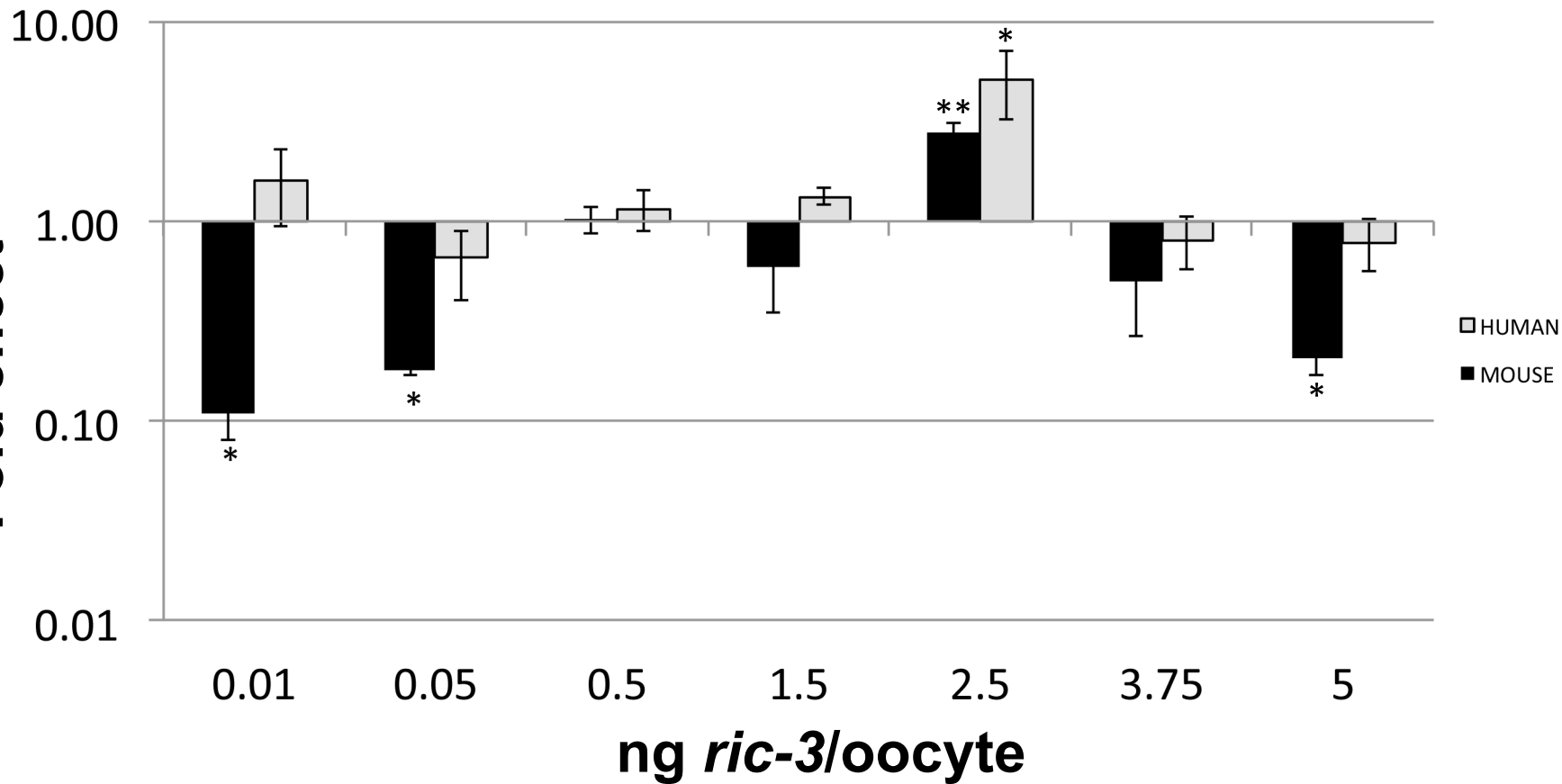

Supplement: Additional file 2: Figure S2. — Effects of FL human RIC-3 vs. mouse FL on α7 nAChR functional expression in X. leavis oocytes. Results were normalized to currents recorded in oocytes expressing the respective receptors in the absence of RIC-3 in the same experiment. Each bar represents 10–20 oocytes from 2 to 3 independent X. laevis. The y-axis ordinates are on a log scale. * indicates a p value of less than 0.05; ** indicates a p value of less than 0.01. (PDF 714 kb) [file 13041_2016_231_MOESM2_ESM.pdf]
